# Do single‐case experimental designs lead to randomised controlled trials of cognitive behavioural therapy interventions for adolescent anxiety and related disorders recommended in the National Institute of Clinical Excellence guidelines? A systematic review

**DOI:** 10.1002/jcv2.12181

**Published:** 2023-07-04

**Authors:** Tom Cawthorne, Anton Käll, Sophie Bennett, Elena Baker, Emily Cheung, Roz Shafran

**Affiliations:** ^1^ Royal Holloway University of London London UK; ^2^ Camden and Islington NHS Foundation Trust London UK; ^3^ Department of Behavioural Sciences and Learning Department of Biomedical and Clinical Sciences Linköping University Linköping Sweden; ^4^ UCL Great Ormond Street Institute of Child Health London England; ^5^ Kent and Medway NHS and Social Care Partnership Trust Gillingham UK

**Keywords:** adolescence, anxiety, CBT, single‐case experimental design (SCED)

## Abstract

**Background:**

Although Cognitive Behavioural Therapy (CBT) is effective for 60% of adolescents with anxiety disorders, only 36% are in remission post‐intervention. This indicates that more effective treatments are needed which should be reflected in the NICE guidelines. We hypothesised that Single‐case experimental designs (SCEDs) may provide a framework for accelerating the development of novel interventions. The primary purpose of this review was to investigate whether SCEDs are currently followed by randomised controlled trials (RCTs) of CBT intervention for adolescent anxiety disorders named in the NICE guidelines. The secondary objective was to investigate whether using SCEDs prior to RCTs could be a helpful approach.

**Method:**

For the primary search of SCEDs five databases were used (PsycINFO, PubMed, PsycArticles, Web of Science and ProQuest). Nineteen articles met eligibility criteria including a total of 107 participants. For the secondary search of RCTs named in the NICE guidelines for adolescent anxiety disorders 53 articles met inclusion criteria and were included in the systematic review.

**Results:**

The 19 SCED studies included in the review were conducted with participants with a diverse range of anxiety disorders and across a range of CBT formats. Two of the SCEDs were followed by RCTs, but neither of these were named in the NICE guidelines for anxiety disorders. All of the SCEDs identified were rated as low quality with none meeting the criteria for the highest or second highest quality rating. From the secondary searches, none of the RCTs named in the NICE guide were preceded by SCEDs.

**Conclusions:**

It was concluded that currently SCEDs were not followed by RCTs of CBT interventions named in the NICE guidelines for adolescent anxiety disorders. However, it was suggested that SCEDs may provide an important framework for the development of more effective interventions for adolescents with anxiety.

**Key points**

- Cognitive Behavioural Therapy (CBT) is effective for 60% of adolescents with anxiety disorders, but only 36% are in remission post‐intervention. This suggest that more effective treatments are needed.
- Historically it takes an average of 7 years from grant application to randomised controlled trials (RCT) publication and 17 years for research evidence to reach clinical practice.
- We suggested that SCEDs could be used to identify interventions most likely to be efficacious and prioritise them for RCT funding, accelerating the process of treatment development.
- We found that currently SCEDs are not being routinely used prior to RCTs of CBT interventions for adolescent anxiety disorders named in the NICE guidelines.
- However, we found evidence that SCEDs were being used to provide high‐quality evidence for a diverse range of CBT interventions for adolescent anxiety. This included groups of young people for whom RCT evidence is unavailable.
- This study demonstrated that using SCEDs prior to RCTs of CBT interventions for adolescent anxiety is a helpful approach and could lead to more effective treatments for young people with anxiety disorders. This finding has significant implications for research policy and clinical practice.

## INTRODUCTION

Cognitive Behavioural Therapy (CBT) is an evidence‐based treatment for anxiety and related disorders in children and adolescence (Baker et al., 2021), with approximately 60% of young people reporting symptom improvement following intervention (James et al., 2013). Accordingly, CBT is recommended in the NICE guidelines for the treatment of social anxiety disorder (SAD), post‐traumatic stress disorder (PTSD), obsessive‐compulsive disorder (OCD) and body dysmorphic disorder for children and adolescence (NICE, 2005; NICE, 2013; NICE, 2018).

However, adolescents are typically underrepresented in treatment outcome studies and only 36% go into remission from their primary anxiety disorder after CBT treatment (Baker et al., 2021), compared to 49.4% of young people across childhood (James et al., 2020). There are inconsistent findings regarding whether adolescents have poorer treatment outcomes than younger children (Bennett et al., 2013; Sauter et al., 2009). However, it has been suggested that if outcomes in research trials are comparable this may be due to expert trial therapists adapting intervention protocols for the adolescent population, with this not being the case in routine clinical practice (Bennett et al., 2013). This is concerning as adolescent anxiety disorders are associated with poor psychosocial outcomes and longitudinally predictive of adult mental health problems (Kendall et al., 2004; Woodward & Fergusson, 2001).

There are several possible reasons for the modest efficacy of CBT for adolescent anxiety disorders, including lack of engagement (Sauter et al., 2009), the biopsychosocial changes observed during this period (Pfeifer & Allen, 2021), such as impairments in fear expression and extinction (Waters et al., 2017), higher rates of comorbid mood disorders and school refusal, and more severe anxiety disorders at baseline compared to younger children (Waite & Creswell, 2014). These difficulties may then be further exacerbated by a lack of specific treatment guidelines (e.g., NICE, 2011).

### NICE guideline development

The NICE guidelines are developed and updated using a clear set of principles (Kelly et al., 2010; NICE, 2014). The quality of the different types of evidence can be visualised as a hierarchy (NICE, 2014). Randomised Controlled Trials (RCTs) are positioned as the ‘gold standard’ for intervention research (Cartwright, 2010). However, despite the methodological rigour of the approach, RCTs present with a significant financial burden (Speich et al., 2018). This high financial cost and the methodological complexity of RCTs limits the number of treatments that can be evaluated, resulting in an average of 7 years from grant application to RCT publication (Riley William et al., 2011) and approximately 17 years for research evidence to reach clinical practice (Morris et al., 2011).

Alternative research methodologies can be used to complement the RCT approach. These designs can identify the interventions that are most likely to be efficacious, which can then be prioritised to accelerate treatment development. Currently, case series are commonplace in the evaluation of psychological interventions. They have been used as precursors to RCTs of CBT interventions for adult anxiety disorders named in the NICE guidelines (Ehlers et al., 2005; Wells & Papageorgiou, 2001), and used in the development of adolescent‐specific protocols of anxiety disorders (Leigh & Clark, 2016), which are now being tested in RCTs (Leigh & Clark, 2019). However, due to their uncontrolled nature case studies have low levels of validity and high level of bias have been identified. This limits the generalisability of results and causal inferences cannot be made (Nissen & Wynn, 2014).

An alternative to the case series is the single case experimental design (SCED) (Smith, 2012). In the SCED approach the dependent variable is repeatedly measured over multiple phases alongside the manipulation of the independent variable, as the intervention is introduced and withdrawn‐see Smith (2012) for a review. SCEDs are a methodologically rigorous alternative to other group designs and allow for causal inferences to be made, whilst also giving the idiographic detail and richness commonly associated with case studies (Kazdin, 2019). Specialist reporting guidelines, risk of bias tools, and specialist methods of visual and statistical analysis have also been developed (Tate et al., 2013). Within these guidelines SCED quality is increased via mechanisms that improve validity, such as control conditions, randomisation, frequent sampling of target behaviour across phases, blinding, measuring adherence, use of standardised measures of the target problem alongside frequent sampling of the target behaviour, and accurate and detailed reporting within the study methodology.

Similarly to case studies, SCEDs are also relatively low‐cost and can be conducted on a far smaller scale than RCT designs. The methodological strengths of the SCED approach also means that they are considered higher quality evidence than case series on the NICE hierarchy and of sufficient quality to influence guidelines development (Kelly et al., 2010; NICE, 2014).

### Summary and aims of this systematic review

More effective CBT interventions are needed for adolescent anxiety disorders, and these should be reflected in the NICE treatment guidelines. It is hypothesised that SCEDs could be utilized to identify interventions most likely to be efficacious and prioritise them for RCT funding to accelerate intervention development. As SCEDs are of sufficient quality to be used as the basis for treatment recommendations (Kelly et al., 2010; NICE, 2014), their evidence can also be used for treatment planning when RCTs are unavailable. This approach could then lead to the faster development of interventions and updated NICE guidelines for adolescent anxiety disorders in order to improve treatment outcomes.

The purpose of this systematc review is:

A) To investigate whether SCEDs were currently followed by RCTs of CBT interventions named in the NICE guidelines for child and adolescent anxiety disorders. The primary method will be a systematic search of databases for SCEDs of CBT interventions for adolescent anxiety disorders. This will be supplemented by a secondary backwards search of RCTs currently cited in the NICE guidelines. These results will then be cross‐referenced with the identified SCEDs.
B) To assess whether it would be useful to use SCEDs prior to RCTs by answering the following questions:
  1) Are there high‐quality SCEDs of CBT interventions for adoelscent anxiety disorders in the literature?
  2) Do SCEDs of CBT interventions for adolescent anxiety disorders lead to subsequent RCTs of the intervention?
  3) Is SCED study quality predictive of whether there is a subsequent RCT?
  4) Are SCED results predictive of subsequent outcomes in RCTs, where an RCT has followed a SCED?

## METHODS

The conduct and reporting of this systematic review is in accordance with the Preferred Reporting Items for Systematic Reviews and Meta‐Analyses (PRISMA) statement (Page et al., 2021). The protocol for the review was registered with the PROSPERO database (https://www.crd.york.ac.uk/prospero/) on 28.04.22. Registration number: CRD42022320071.

### Part 1: Systematic search of SCEDs

#### Eligibility criteria

This review searched for studies reporting single‐case experimental designs (SCEDs) of CBT interventions for adolescent anxiety disorders.

#### Inclusion/exclusion criteria:

1. The study had to be published in English, or with an English translation available.
2. Peer‐reviewed journal articles and unpublished manuscripts were included. The rationale for the latter was that SCEDs may be used in early stage treatment development and therefore not published in peer‐reviewed journals.
3. The study reports the results of a Single‐Case Experimental Design (SCED) as defined by the methodology (Smith, 2012). SCEDs are characterised by specific methodological criteria including repeated measurement of the dependent variable over a minimum of two phases alongside manipulation of the independent variable (Kazdin, 2019). There was no minimum requirement for the number of times the dependent varibale needed to be repeated in each phase, although the number of observations was reflected in the study quality rating (Tate et al., 2013).
4. The study population is adolescents. As with other meta‐analyses of adolescent anxiety disorders (Baker et al., 2021), the adolescent period was defined as 11–18. This is based on 11 being the average age that external signs of puberty become apparent and 18 being the legal age of adulthood, when child and adolescent mental health services end in most countries. Furthermore, 11–18 is the period of secondary education, so young people in this age range have similar social and educational demands and experiences. Manuscripts were therefore included where the mean age was within the 11–18 age range. If the mean age was not reported and could not be calculated, studies were included if the middle of the age range was between 11 and 18.
5. The intervention is based within a Cognitive Behavioural Therapy (CBT) framework as defined by the study team. Meaning that any interventions where the authors used the term “CBT”, “cognitive behavioural therapy” or similar terminology were included. Where this was unclear the description of the intervention was reviewed to ascertain whether it was based upon cognitive behavioural therapy principles (Butler et al., 2006).
6. The outcome of the study is symptoms of anxiety. This could include any validated anxiety measure, a visual analogue scale, or idiographic measure of anxiety as commonly used in SCEDs (Kazdin, 2019; Smith, 2012). PTSD, OCD, BDD and hoarding were included under the umbrella of “anxiety related disorders” as there are significant anxiety components underlying each disorder, with avoidance being a key maintenance factor as with other anxiety disorders.

#### Information sources

PsycINFO, PubMed, PsycArticles, Web of Science and ProQuest were used as databases. These included searches of the grey literature and unpublished dissertations and PhD theses were included. The search was completed on April 12^th^ 2022. The references of included papers were systematically searched to identify further studies meeting eligibility criteria.

The SCEDs included within the systematic review after screening was completed were then entered into the databases and a ’cited by’ search was conducted to identify whether there was a subsequent RCT. The rationale for this being that any RCT would cite the preceding SCED. Due to the average of 7 years from grant application to RCT publication (Riley William et al., 2011), it was also examined whether there was a published protocol paper for any RCTs that are ongoing.

#### Search strategy

The following search terms were entered (see Table 1). The Boolean operator “AND” was used to combine concepts in order to make the results more relevant to the questions of this systematic review. We set the searches to identify articles where the terms were found anywhere within the text. We searched for articles published from database inception until April 12^th^ 2022 and we reviewed all papers that were available.

**TABLE 1:** Search terms entered for the database searches.

Concept	Search terms
SCED	“SCED” OR “single‐case” OR “single case” OR “single case experimental design” OR “single‐case experimental design"
Adolescence	“Child*” OR “adolescen*” OR “teenager” OR “young person” OR “young people” OR “youth”
Anxiety	“Anxiety” OR “anxious” OR “social anxiety disorder” OR “SAD” OR “PTSD” OR “post traumatic stress disorder” OR “post‐traumatic stress disorder” OR “OCD” OR “obsessive compulsive disorder” OR “obsessive‐compulsive disorder” OR “panic” OR “agoraphobi*” OR “generalised anxiety disorder” OR “generalized anxiety disorder” OR “GAD” or “body dysmorphic disorder”
CBT	“CBT” OR “cognitive behavioural therapy” OR “cognitive therapy”

#### Selection process

**FIGURE 1:**
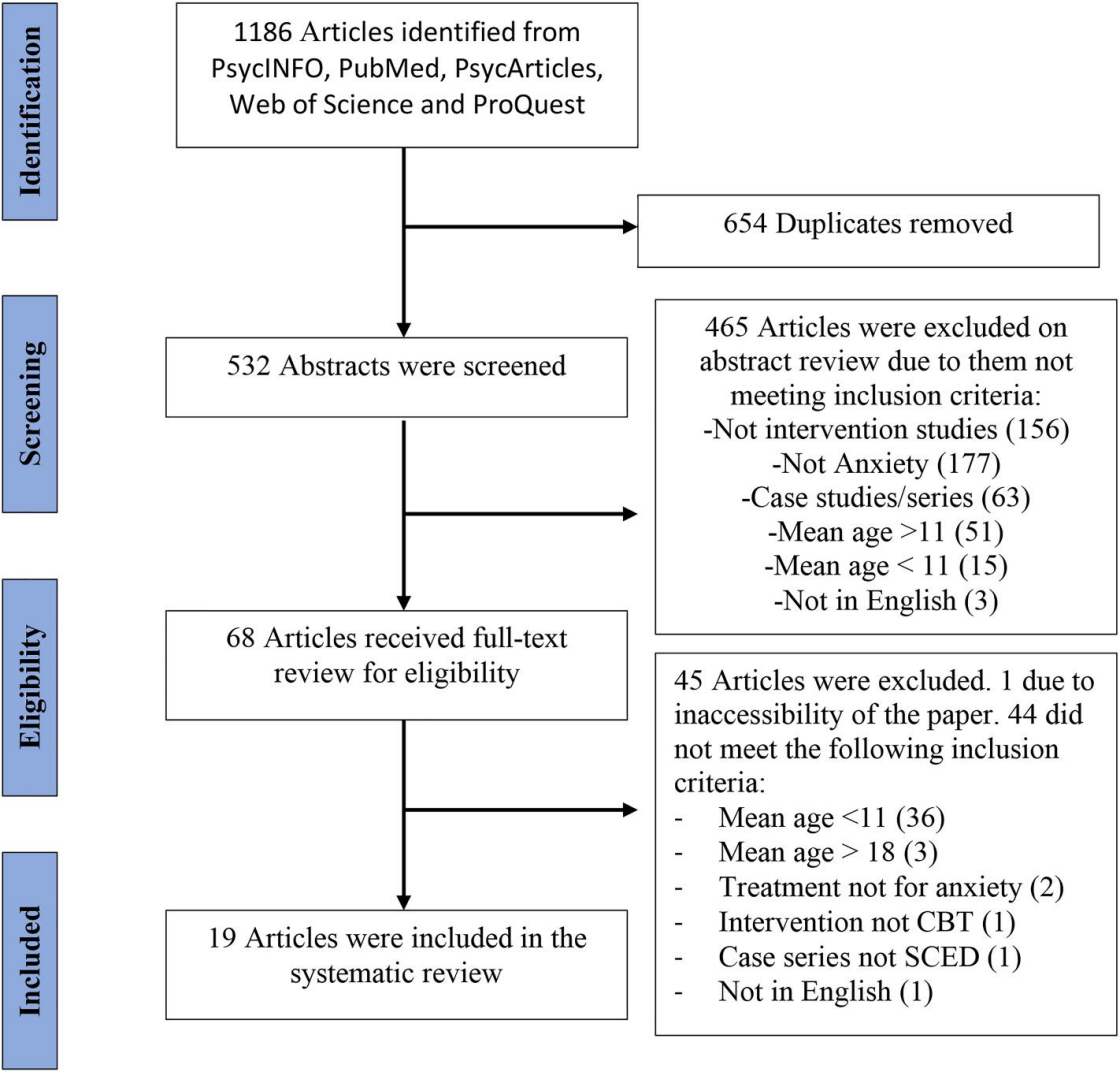
PRISMA flowchart for the selection process of the SCED studies.

Articles were identified, screened and then assessed following PRISMA guidelines (Page et al., 2021). See Figure 1 for flowchart. Rayyan was used as the systematic review tool (Ouzzani et al., 2016). The searches were completed by the first author who then assessed and removed 654 duplicate results. The author then completed the initial screening of the abstracts of the 533 publications identified. 10% (Knight et al., 2019) of the studies were selected at random using a random number generator (www.random.org) and examined by a second independent researcher. The inter‐rater agreement was 96.25%. Any conflicts of opinion regarding study eligibility were discussed until a consensus reached. Sixty‐eight articles then received a full‐text review for eligibility. Nineteen articles were included in the systematic review which included a total of 107 participants. 10% (Bennett et al., 2013) of these articles were selected at random using a random number generator (www.random.org) and were screened by the second independent researcher for suitability, with inter‐rater agreement of 100%. Of the 45 excluded papers, 1 was excluded as the paper could not be accessed and the author did not respond to an email requesting the paper. Fourty‐four papers were excluded as they did not meet eligibility criteria.

#### Data extraction

Data extraction was completed by the first author. A headed table was used to facilitate the extraction of information from the manuscript texts. All extracted data was checked by a second researcher (EB) in order to minimise the probability of errors. Socio‐demographic and clinical characteristics were extracted from the eligible studies. Whether the SCEDs were followed by an RCT was also determined by doing a ‘cited by’ search in the databases. Based on the assumption that if there was a subsequent RCT, it would cite the prior SCED. This was reported as a binary outcome (Yes/No). If an RCT was conducted, this was then cross‐referenced with the list of RCTs reported under the ‘Evidence’ section of each of the NICE guidelines for anxiety disorders and it was recorded whether the RCT was named in the NICE guidelines (Yes/No). Whether the results of the SCED was predictive of the RCT outcome was also reported, defined as a binary outcome (Congruent/Incongruent). It was recorded as congruent if both the SCED and RCT reported the same outcome, that is, that the intervention was effective or non‐effective. It was not possible to report and compare effect sizes, as the required information was not included in the SCED studies.

The data extracted included:

a) Participant sample
b) Participant mean age
c) Participant age range
d) Participant gender composition (% male)
e) Sample size
f) Target anxiety disorder
g) Delivery method of CBT intervention (e.g., parent‐led, group etc.)
h) Outcome measure used for repeated measurement
i) Was the SCED followed by an RCT (Yes/No). If so:
  i) Were the results of the SCED predictive of the RCT outcome? (Congruent/Incongruent). Operationalised as whether both reported the same outcome regarding the interventions efficacy. Whether a SCED was classified as efficacious was determined by the results of Tau‐U analysis (Parker et al., 2011) and/or visual inspection (Kazdin, 2019; Lane & Gast, 2014) as consistent with SCED procedure and guidelines (Smith, 2012; Tate et al., 2013).
  ii) Was the RCT named in the NICE guidelines for child and adolescent anxiety disorders (Yes/No)

If data were not reported (e.g., mean age) it was calculated by the primary researcher where possible. If data was not available then this is indicated by ‘– ‘in the table. No effect sizes were calculated as this is not typical within the SCED approach, with the studies also not giving sufficient information to allow for effect size calculation.

#### Study risk of bias assessment

The RoBiNT scale (Tate et al., 2013) was used to assess study quality and risk of bias of the SCEDs included in the systematic review. The tool is a 15‐item measure which is comprised of two subscales, internal validity (7 items) and external validity and interpretation (8 items) with the scoring system using a 3‐point scale (0–2). For each study the internal validity subscale (maximum score 14), external validity and interpretation subscale (maximum score 16), and total score subscale (maximum score 30) was calculated and reported. Psychometric evaluation of the scale showed evidence of construct validity, with excellent levels of inter‐rater reliability, with interclass correlational coefficients of 90% (Tate et al., 2013). For level 1 (the highest quality rating), studies must achieve scores of two on items 1 (experimental design), 2 (randomisation) and 3 (sufficient sampling) on the internal validity subscale. Level two studies need to achieve a score of two on items 1 and 3. Level 3 studies need to achieve a score of one on items 1 and 3. Level 4 studies are SCEDs with serious design flaws that did not meet the above scoring criteria. While finally level 5 are non‐experimental single case designs (e.g., case series). The first author completed the risk of bias assessment for each of the SCEDs included in the systematic review. A second independent researcher then completed the risk of bias assessment for 10% of the papers selected by a random number generator. Inter‐rater reliability was 91%, which is comparable to the measure validation paper (Tate et al., 2014).

### Part 2: Backwards searches of NICE guidelines

#### Eligibility criteria

The review also searched for Randomised Controlled Trials (RCTs) named in the NICE guidelines for anxiety disorders. The search included all RCTs named in the ‘evidence’ section of the relevant NICE guidelines as of April 12^th^ 2022.

Inclusion/exclusion criteria:

1. The study had to be published in English, or with an English translation available.
2. The study reports a randomised controlled trial (RCT)
3. The study was conducted with adolescents. Defined as ages 11–18 (as above).
4. The intervention is based within a Cognitive Behavioural Therapy (CBT) framework as defined by the study team.
5. The study is listed under the “Evidence” section for one of the NICE guidelines for generalised anxiety disorder (GAD) and panic disorder (NICE, 2011), PTSD (NICE, 2018), SAD (NICE, 2013) and OCD and BDD (NICE, 2005). All NICE guidelines for anxiety disorders were included regardless of whether there were specific guidelines for children and adolescence in case relevant RCTs were cited as evidence for general guideline development.

#### Information sources and search strategy

The source of the information was the ‘Evidence’ sections of the NICE guidelines for anxiety disorders. This included the guidelines for GAD and panic disorder (NICE, 2011), PTSD (NICE, 2018), SAD (NICE, 2013), and OCD and BDD (NICE, 2005). All the RCTs for each of the anxiety disorders were systematically collated by a student research assistant.

#### Selection process

**FIGURE 2:**
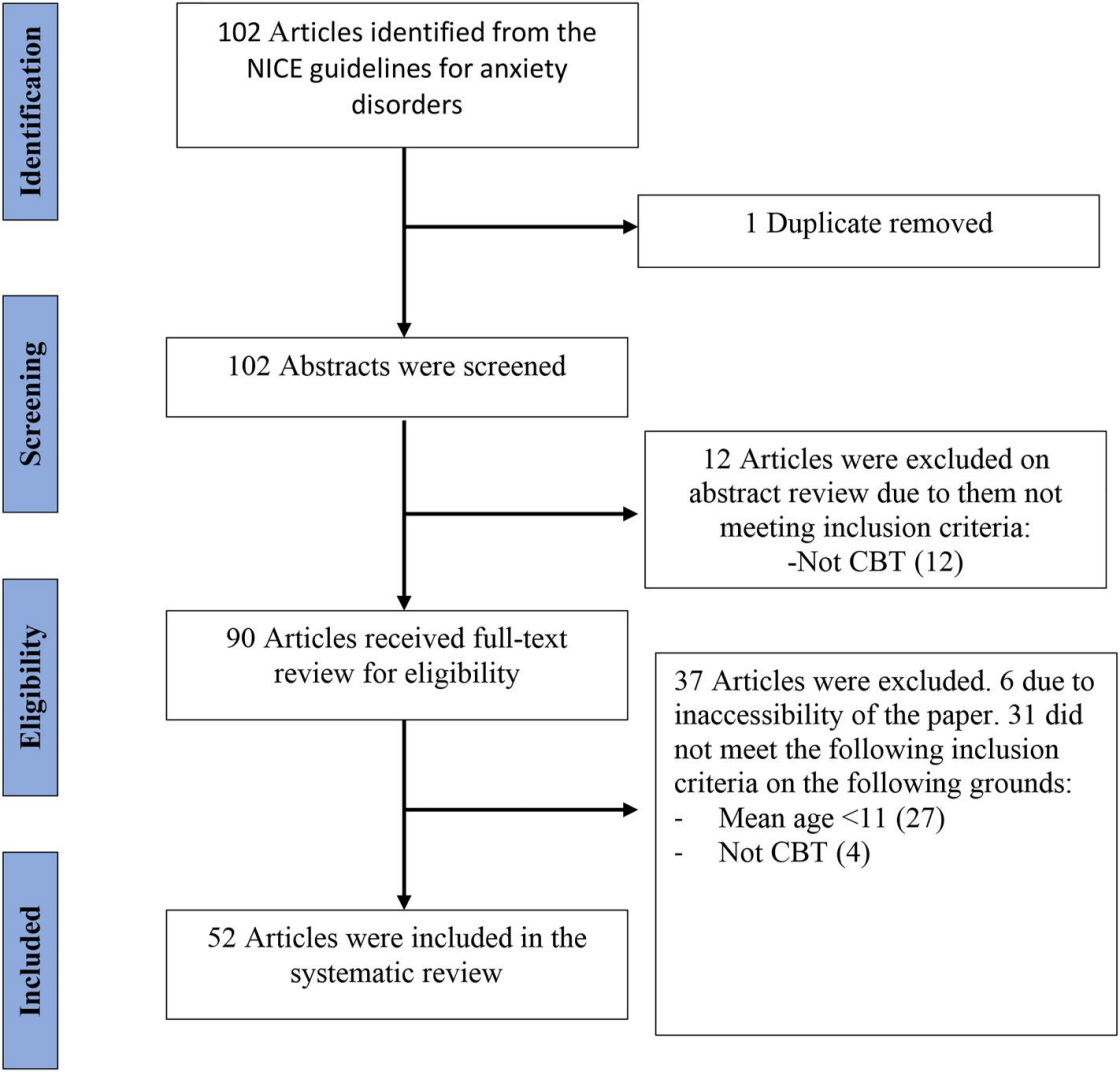
PRISMA flowchart for the selection process of the RCT studies.

The RCTs were then systematically screened to identify eligible studies. See Figure 2. The search was completed by the first author and 1 duplicate was removed. The author then completed the initial screening of the abstracts, and 90 publications were identified. 10% of the studies were selected at random using a random number generator (www.random.org) and examined by a second independent researcher. The inter‐rater agreement was 100%. Ninety articles then received a full‐text review for eligibility, with 52 articles meeting criteria for inclusion in the systematic review. 10% of these articles were selected at random using a random number generator (www.random.org) and were screened by the second independent researcher for suitability, with inter‐rater agreement of 100%. 37 Articles were excluded. Six due to inaccessibility and 31 not meeting inclusion criteria.

#### Data extraction of RCTs named in the NICE guidelines for anxiety disorders

Data were extracted and brief socio‐demographic and clinical characteristics were reported. Whether there was a previous SCED was determined by a search of the manuscript, on the assumption that if there was it would be cited. The characteristics reported included:

A) Participant mean age
B) Participant age range
C) Target anxiety disorder
D) Was there a previous SCED (Yes/No)

#### Data synthesis and analysis

The studies included within the systematic review were synthesised before being summarised narratively. This approach follows published guidelines (Popay et al., 2006). The narrative synthesis was conducted to answer whether SCEDs were followed by RCTs of CBT interventions and whether these were named in the NICE guidelines for child and adolescent anxiety disorders. It was also examined whether using SCEDs prior to RCTs in this population would be a useful approach.

Therefore, the synthesis covered the following categories:

1) Whether SCEDs of CBT interventions for adolescent anxiety disorders are followed by RCTs, and whether these RCTs are named in the NICE guidelines.
2) Whether it would be useful to use SCEDs prior to RCTs by answering the following questions:
  a) Are SCED results predicitve of subsequent outcomes in RCTs?
  b) Are there high‐quality SCEDs of CBT interventions for adoelscent anxiety disorders in the literature?
  c) Is SCED study quality predictive of whether there is a subsequent RCT?

It was not expected that there would be a sufficient number of homogeneous studies to conduct a meta‐analysis. Within the SCED approach it is not typical to report effect sizes and so this was not included, with the studies also not giving sufficient information to allow effect size calculation.

## RESULTS

In total 19 SCED studies met inclusion criteria. Table 2 provides an overview of the study characteristics, with further information on study quality in Appendix S1. The studies were published between 1989 and 2020. The sample sizes ranged from 1 to 17 (mean = 5.6) with 6/19 (31.6%) including just 1 participant. In total 107 participants were included across the studies. The grand mean age across the sample was *M* = 13.70% and 40.3% of the total sample were male. The target anxiety disorder of the interventions varied, GAD (*n* = 2), OCD (*n* = 3), PTSD (*n* = 4), Phobia (*n* = 1) and Hoarding (*n* = 1). Several of the studies did not specify a specific anxiety disorder and used the term “anxiety” or “childhood anxiety” more generally (*n* = 3) whilst others reported on participants with multiple comorbid anxiety disorders (*n* = 5).

**TABLE 2:** Characteristics of SCEDs of CBT interventions for adolescent anxiety disorders.

Author, year	Participant sample	Mean age	Age range	Gender (% male)	Sample size	Target anxiety disorder	Type of CBT intervention	Repeated measure	RoBiNT total (quality level)	Was the SCED followed by an RCT?	Were SCED results predictive of RCT outcome?	Was the RCT named in the NICE guidelines?
Bernstein (2010)	School setting	16	11–18	50	4	Non‐specified anxiety	School‐based modular CBT	Self‐reported fear ladder ratings	16 (3)	No	N/A	N/A
Cowles and Davis (2017)	Community clinic	16	16	0	1	PTSD	Trauma‐focussed CBT	The child PTSD symptom scale (Foa et al., 2001)	11 (4)	No	N/A	N/A
Farrell et al. (2016)	Community‐ self‐referred	13.6	11–16	60	10	OCD	Individual exposure response prevention (ERP) with *E* therapy maintenance	Children's Yale‐Brown obsessive‐compulsive scale–parent report (Freeman et al., 2011)	15 (4)	Yes (Farrell et al., 2022)	Congruent	No
Feather and Ronan (2009)	Community clinic‐ maltreated children	11.6	9–13	50	8	PTSD	Trauma‐focussed CBT	Children's post‐traumatic stress reaction index (Frederick et al., 1992)	15 (4)	No	N/A	N/A
Grefe (2011)	Community clinic‐ experienced sexual or physical assault	14.7	13–17	0	9	PTSD	Trauma‐focussed CBT, incorporating DBT, parent training and motivational interviewing	Child PTSD symptoms scale (Foa et al., 2001), BASC‐2 (Kamphaus, 2014) life problems inventory (Rathus et al., 2015)	15 (4)	No	N/A	N/A
Hains et al. (1997)	Community clinic‐ cystic fibrosis	13.8	13–15	60	5	Non‐specified anxiety	Individual CBT	The state‐trait anxiety inventory for children (Spielberger et al., 1973)	11 (4)	No	N/A	N/A
Heard et al. (1992)	Community clinic	13.3	12–15	0	3	Phobia	Behavioural exposure	Self and parent‐rated frequency of problem behaviour	13 (4)	No	N/A	N/A
Houghton et al. (2017)	Community clinic‐ ADHD	14.2	13–16	70	10	GAD	Group CBT	Self‐reported anxiety on a 1–10 visual analogue scale	13 (3)	No	N/A	N/A
Kane and Kendall (1989)	Community clinic	11.5	10–13	25	4	Phobia or overanxious disorder	Individual CBT	The state‐trait anxiety inventory for children (Spielberger et al., 1973)	17 (3)	Yes (Kendall, 1994; Kendall et al., 1997)	Congruent	No
Knight et al. (2019)	Community clinic	15	15	0	1	Hoarding	Individual CBT	Outcome rating scale (Miller et al., 2003)	11 (4)	No	N/A	N/A
March et al. (1998)	School setting	‐	9–15	33	17	PTSD	Group trauma‐focussed CBT	Child and adolescent trauma survey (Suliman et al., 2005)	7 (4)	No	N/A	N/A
Neil et al. (2017)	Community clinic‐ autism + OCD	11	11	100	1	OCD	Functional behaviour‐ based CBT	Parent OCB rating scale (Vause et al., 2017)	16 (4)	No	N/A	N/A
Ollendick (1995)	Community clinic	15	13–17	25	4	Panic disorder and agoraphobia	Individual CBT	Self‐reported frequency of panic attacks, agoraphobic avoidance and self‐efficacy for coping with panic attacks on a 1–5 scale	12 (4)	No	N/A	N/A
Petoskey (2015)	Community clinic‐ autism	18	18	100	1	Non‐specified anxiety	School based CBT	The multidimensional anxiety scale for children—second edition (Wei et al., 2014)	13 (4)	No	N/A	N/A
Roberts‐Collins (2016)	Community clinic	15	15	0	1	Health anxiety and OCD	Individual CBT	RCADS OCD subscale (Chorpita et al., 2000)	11 (4)	No	N/A	N/A
Sieberg et al. (2011)	School setting/Community clinic‐ recurrent abdominal pain	11.5	10–12	25	8	Separation anxiety, social phobia, specific phobia and GAD	Family based CBT	Anxiety disorders interview schedule, parent and child versions (Silverman & Nelles, 1988)	12 (4)	No	N/A	N/A
Sukhodolsky et al. (2013)	Community clinic	13	9–14	83	6	OCD	ERP + parent management training	Children's Yale‐Brown obsessive‐compulsive scale–parent report (Freeman et al., 2011)	15 (4)	No	N/A	N/A
Wahlund et al. (2020)	Community clinic	14.6	13–17	85	13	GAD	Online CBT intervention	Brief Penn state worry questionnaire (Topper et al., 2014)	9 (4)	No	N/A	N/A
Waldron et al. (2018)	Community clinic	16	16	0	1	Social anxiety, panic and agoraphobia	Individual CBT	RCADS social anxiety subscale (Chorpita et al., 2000)	10 (4)	No	N/A	N/A

Fifty‐Two RCTs also met the inclusion criteria of the backwards searches and are reported in Appendix S2. The studies were published between 2000 and 2017. The mean ages of study participants ranged from 11 to 17. The RCTs primarily focussed on PTSD (*n* = 45) with the others being RCTs of CBT interventions for SAD (*n* = 5), childhood anxiety (*n* = 1) and OCD (*n* = 1).

**Are SCEDs of CBT interventions for adolescent anxiety disorders followed by RCTs? Are SCED results predictive of RCT outcomes? And are these RCTs are named in the NICE guidelines?**

Of the 19 SCEDs included in the review, two had subsequent RCTs (Farrell et al., 2016; Kane & Kendall, 1989); 17 did not. In both cases these RCTs following the SCEDs found evidence of intervention efficacy, congruent with the prior SCED. Both Kendall RCTs (Kendall, 1994; Kendall et al., 1997) found a significant reduction in parent and self‐reported anxiety at post‐intervention compared to treatment as usual for young people with any primary anxiety disorder, which was maintained at 1‐year follow up. In contrast Farrell et al. (2022), reported on an RCT comparing a CBT exposure‐based intervention for OCD to the same intervention augmented with the medication D‐cycloserine, finding improvements in both groups at post‐intervention, although no significant difference between conditions. However, these RCTs were not named in the relevant NICE guidelines for child and adolescent anxiety disorders (see Appendix S2). None of the other SCEDs had been followed by RCT protocol papers indicating an ongoing RCT. For the second part, none of the 52 RCT studies identified through the backwards searches of the NICE guidelines reported that they were preceded by a SCED.

### SCED quality appraisal

**Are there high‐quality SCEDs of CBT interventions for adolescent anxiety disorders in the literature?**

The total RoBiNT (Tate et al., 2013) score and Quality Level was reported. For level 1 (the highest quality rating), studies must achieve scores of two on items 1 (experimental design), 2 (randomisation) and 3 (sufficient sampling) on the internal validity subscale. Level two studies need to achieve a score of two on items 1 and 3. Level 3 studies need to achieve a score of one on items 1 and 3. Level 4 studies are SCEDs with serious design flaws that did not meet the above scoring criteria. While finally level 5 are non‐experimental single case designs (e.g., case series). The mean RoBiNT score was 12.74 (SD = 2.66) with scores ranging from 7 to 17 as shown in Appendix S1. None of the studies achieved a quality rating of level one or level two, 4 met the criteria for level 3 and 15 for level 4. No studies met criteria for level 5 as non‐experimental single case designs were excluded from the study. See Appendix S1 for quality summary.

**Is SCED study quality predictive of whether there is a subsequent RCT?**

No clear evidence of a relationship between SCED quality and recency of publication was identified. Within the internal validly subscale, which is used to calculate the RoBiNT quality level, the lack of blinding and ratings of interrater agreement and treatment adherence were significant areas of weakness. The two studies which had subsequent RCTs had RoBiNT ratings of 15 (Kane & Kendall, 1989) and 17 (Farrell et al., 2016) and both received a Level 4 for study quality. As only two of the SCEDs had RCTs it was not possible to do an independent *T*‐test to further examine the relationship between study quality and whether there was a subsequent RCT.

## DISCUSSION

This systematic review identified 19 SCEDs of CBT interventions for adolescent anxiety disorders that were published over a 31‐year period. Nineteen eligible studies are relatively few, suggesting that the methodology has yet to become commonplace for the evaluation of CBT interventions for adolescent anxiety disorders. However, 13 of the 19 studies identified were published since 2010, which may indicate that the approach is beginning to increase in popularity.

Of the 19 SCEDs, only two resulted in RCTs of the intervention (Farrell et al., 2016; Kane & Kendall, 1989); with Kane & Kendall (1989) having two subsequent RCTs (Kendall, 1994; Kendall et al., 1997). Both SCEDs reported the interventions to be efficacious, with this being congruent with the finding of the RCTs. However, as the relationship between SCED and RCT outcomes could only be examined for two studies it could not be confidently determined whether SCED results are predictive of subsequent RCT outcomes. This is an important area for future research.

Another finding was that neither of the RCTs that were conducted following SCEDs were named in the NICE guidelines. However, there is a recent addition to the literature (Farrell et al., 2022) which may be included in the future, as the NICE guidelines for OCD has not been updated since 2013 (NICE, 2013). The backwards searches of the RCTs currently named in the NICE guidelines also found that none were preceded by a SCED of the intervention (Appendix S2). Therefore, it can be concluded that despite the methodological rigour of the SCED approach (Kazdin, 2019), currently SCEDs are not typically used prior to RCTs of evidence‐based CBT interventions for adolescent anxiety disorders named in the NICE guidelines.

This finding is surprising due to the clear rationale for this approach and because one of the SCEDs identified (Kane & Kendall, 1989) preceded ‘Coping Cat’ (Kendall, 1994), which is among the most evidenced CBT interventions for pre‐adolescent children with anxiety (Lenz, 2015), despite not mentioned in the NICE guidelines. One reason for this may be that currently NICE guidelines for child and adolescent GAD and panic disorder are lacking. Therefore, this omission is a limitation of the NICE guidelines, rather than of the SCED approach.

There are several possible reasons for the finding that SCEDs are not typically being followed by RCTs, including the low quality scores identified on the RoBiNT scale. However, caution should be taken when interpreting these ratings. Although the RoBiNT is specifically designed for SCED studies, historically SCEDs have primarily been used within the field of behavioural and educational interventions (Smith, 2012), rather than psychotherapies like CBT, thus limiting the generalisability of several of the items. For instance, to achieve a Level 1 or 2 quality rating score, studies had to score 2‐points on the experimental design item of the measure. To achieve a 2‐points rating for design the study must as a minimum be an ABAB with 4 phases; concurrent multiple‐baseline design (MBD) with 6 phases, 3 tiers; alternating‐treatments design (ATD) with 4 sets of alternating sequences or a changing‐criterion design (CCD) with 4 steps (Tate et al., 2013). As the purpose of CBT treatment is to use therapy to equip individuals with skills that they can then utilise outside of the therapy room during their day‐to‐day life it is more challenging to implement repeated alternating or changing criterion designs, as participants may continue to use skills learnt in one IV condition once they move in to another phase of the study. Another possible reason for the lack of SCEDs preceding RCTs may be the limited dissemination of the approach compared to alternative methodologies. As 13 of the 19 identified SCEDs were published since 2010, it may that the use of SCEDs for CBT interventions of anxiety disorders in adolescence is becoming more commonplace. However, as there is an average of 7 years between grant application and RCT publication (Riley William et al., 2011) it would be expected that there would be a delay between SCED publication and subsequent RCT evaluation. Although it is noted that no RCT protocol papers were also identified.

Although this review found that SCEDs are not routinely used prior to RCTs of CBT interventions for adolescent anxiety disorders, there was evidence to suggest that this approach could be helpful. The SCEDs identified targeted a range of anxiety difficulties. This included CBT therapy for populations that have not yet had interventions evaluated in RCTs, for example, adolescent hoarders (Knight et al., 2019), those for whom NICE guidelines are currently unavailable, for example, adolescents with agoraphobia, panic disorder and GAD (Houghton et al., 2017; Ollendick, 1995; Wahlund et al., 2020) and young people with multiple anxiety disorders and dual diagnoses (Houghton et al., 2017; Neil et al., 2017; Roberts‐Collins, 2016; Sieberg et al., 2011; Waldron et al., 2018). This indicates that SCEDs may provide a flexible framework for evaluating interventions for a diverse range of anxiety problems in adolescents. Indicating that despite the methodological limitations of the SCEDs identified, they are still being used to provide higher quality evidence than alternative methodologies, (e.g., case studies). Future intervention research should focus on increasing the quality of SCED evaluation informed by relevant guidelines (e.g., RoBiNT). A particular focus should be on increasing the use of blinding procedures and reporting of interrater agreement and treatment adherence.

### Limitations and areas for future research

A limitation of this review is that there are inconsistencies in the terminology used within the literature to describe the SCED approach. Therefore, it is possible that there may be some SCEDs not identified during the searches that would meet the criteria for this review. A second limitation was that inter‐rater percentage agreement was used instead of Cohen's Kappa due to the small sample size. Furthermore, the main finding that SCEDs are not used prior to RCTs of CBT interventions named in the NICE guidelines for adolescent anxiety disorders was based upon searches of the current literature. However, on average there is a 7 years delay from RCT grant application to publication (Riley William et al., 2011). As nine of the 19 SCEDs were published within the last 7 years, it may be beneficial to replicate this review in the future. A further limitation was that although the literature search included protocol papers or ongoing trials, we did not search trial registries. This study also focussed on CBT interventions for adolescent anxiety disorders. Therefore, caution should be taken when extrapolating the findings to other ages, psychological interventions, or mental health difficulties. Future reviews should investigate the role of SCEDs in intervention development for these populations. This review also concluded that whilst SCEDs are currently not being typically used prior to RCTs this approach may be helpful. Further research is needed to investigate barriers to this approach with a sufficient sample to allow for adequately powered statistical analysis. A final limitation was that the included SCED studies reported the gender of participants as binary (male/female) and therefore it is unclear how the interventions can be applied to young people with alternative gender identities.

### Summary

Approximately 60% of adolescents report improvements in anxiety following CBT treatment (James et al., 2013), however only 36% are in remission from their primary anxiety disorder (Baker et al., 2021). Therefore, more effective interventions for adolescents with anxiety and related disorders are needed, which should be reflected in the relevant NICE guidelines. RCTs are considered the ‘gold standard’ for intervention evaluation (Cartwright, 2010). However, they present with a high financial burden (Speich et al., 2018) and do not allow for the idiographic richness of single case designs which is advantageous for treatment development. Currently, RCTs of interventions for anxiety and related disorders are frequently preceded by case series (Ehlers et al., 2005; Leigh & Clark, 2016; Wells & Papageorgiou, 2001). Instead we propose that SCEDs should be used to identify interventions most likely to be efficacious and prioritise these for RCT funding to accelerate treatment development.

The primary purpose of this systematc review was to investigate whether SCEDs of CBT interventions for adolescent anxiety and related disorders were followed by RCTs named in the NICE guidelines. The secondary objective was to assess whether it would be helpful to use SCEDs prior to RCTs in this population. It was found that there were 19 SCEDs of CBT interventions for adoelscent anxiety and related disorders, however only two had subsequent RCTs and these were not named in the NICE guidelines. It was also identified that no RCTs named in the relevant NICE guidelines were preceeded by SCEDs. However, evidence that this approach could be helpful was also found. SCEDs had been used to evaluate a diverse range of interventions for adolecent anxiety and related disorders in a range of real‐world settings. This included populations whereby SCEDs were being used to providde evidence of sufficent quality to be used as the basis for treatment recommendations (NICE, 2014) when RCTs were unavailable. Therefore, it was concluded that SCEDs may provide an important framework for development of more effective interventions for adolecence anxiety disorders.

## AUTHOR CONTRIBUTION

**Tom Cawthorne:** Conceptualization; Data curation; Formal analysis; Investigation; Methodology; Project administration; Resources; Writing – original draft. **Anton Käll**: Conceptualization; Formal analysis; Methodology; Supervision; Writing – review & editing. **Sophie Bennett**: Conceptualization; Investigation; Methodology; Project administration; Supervision; Visualization; Writing – review & editing. **Elena Baker**: Data curation; Formal analysis; Project administration. **Emily Cheung**: Data curation; Project administration. **Roz Shafran:** Conceptualization; Formal analysis; Methodology; Supervision; Writing – review & editing.

## CONFLICT OF INTEREST STATEMENT

The authors have declared that they have no competing or potential conflicts of interest.

## ETHICAL CONSIDERATIONS

Ethical approval was not required for this research review.

## Supporting information

Supporting Information S1

## Data Availability

The data that support the findings of this study are available from the corresponding author upon reasonable request.
