# Supplementary material for: Do single‐case experimental designs lead to randomised controlled trials of cognitive behavioural therapy interventions for adolescent anxiety and related disorders recommended in the National Institute of Clinical Excellence guidelines? A systematic review
Source: JCPP Adv. 2023 Jul 4;3(3):e12181. doi: 10.1002/jcv2.12181 (PMC10501697; doi:10.1002/jcv2.12181)
Supplement: Supplementary file 1 — Supporting Information S1 [file JCV2-3-e12181-s001.docx]

**Supporting Information**

Appendix S1. Scores on each item of the RoBiNT scale

| Author, Year | Design with control | Randomisation | Sampling of Behaviour | Blinding of people involved in the intervention | Blinding of assessor(s) | Interrater agreement | Treatment adherence | Baseline characteristics | Setting | Dependent variable (target behaviour) | Independent variable (therapy/ intervention) | Raw data record | Data analysis | Replication | Generalisation | Internal validity subscale | External validity and interpretation subscale | Total |
| --- | --- | --- | --- | --- | --- | --- | --- | --- | --- | --- | --- | --- | --- | --- | --- | --- | --- | --- |
| Bernstein (2010) | 1 | 2 | 1 | 0 | 0 | 0 | 0 | 2 | 1 | 2 | 2 | 0 | 2 | 2 | 1 | 4 | 12 | 16 |
| Cowles and Davis (2017) | 0 | 0 | 0 | 0 | 0 | 0 | 0 | 2 | 1 | 2 | 2 | 2 | 0 | 0 | 2 | 0 | 11 | 11 |
| Farrell et al. (2016) | 0 | 2 | 0 | 0 | 0 | 0 | 1 | 1 | 1 | 2 | 2 | 0 | 2 | 2 | 2 | 3 | 12 | 15 |
| Feather and Ronan (2009) | 0 | 2 | 0 | 0 | 0 | 0 | 0 | 1 | 1 | 2 | 2 | 2 | 1 | 2 | 2 | 2 | 13 | 15 |
| Grefe (2011) | 0 | 2 | 0 | 0 | 0 | 0 | 0 | 2 | 1 | 2 | 1 | 2 | 1 | 2 | 2 | 2 | 13 | 15 |
| Hains et al. (1997) | 0 | 0 | 0 | 0 | 0 | 0 | 0 | 1 | 1 | 2 | 2 | 2 | 1 | 1 | 1 | 0 | 11 | 11 |
| Heard et al. (1992) | 0 | 0 | 1 | 0 | 0 | 0 | 0 | 2 | 1 | 2 | 1 | 2 | 1 | 1 | 2 | 1 | 12 | 13 |
| Houghton et al. (2017) | 1 | 2 | 2 | 0 | 0 | 0 | 0 | 1 | 1 | 2 | 2 | 0 | 0 | 1 | 1 | 5 | 8 | 13 |
| Kane and Kendall (1989) | 1 | 0 | 1 | 0 | 0 | 0 | 1 | 2 | 2 | 1 | 2 | 2 | 1 | 2 | 2 | 3 | 14 | 17 |
| Knight et al. (2019) | 0 | 0 | 0 | 0 | 0 | 0 | 0 | 2 | 2 | 2 | 2 | 2 | 0 | 0 | 1 | 0 | 11 | 11 |
| March et al. (1998) | 0 | 1 | 0 | 0 | 0 | 0 | 0 | 1 | 1 | 2 | 0 | 0 | 0 | 1 | 1 | 1 | 6 | 7 |
| Neil et al. (2017) | 0 | 0 | 2 | 0 | 0 | 0 | 2 | 2 | 1 | 2 | 2 | 2 | 1 | 0 | 2 | 4 | 12 | 16 |
| Ollendick (1995) | 1 | 0 | 0 | 0 | 0 | 0 | 0 | 2 | 1 | 2 | 2 | 2 | 1 | 0 | 1 | 1 | 11 | 12 |
| Petoskey (2015) | 1 | 0 | 0 | 0 | 0 | 0 | 2 | 2 | 1 | 2 | 2 | 0 | 1 | 0 | 2 | 3 | 10 | 13 |
| Roberts‐Collins (2016) | 0 | 0 | 1 | 0 | 0 | 0 | 0 | 2 | 1 | 2 | 2 | 2 | 0 | 0 | 1 | 1 | 10 | 11 |
| Sieberg et al. (2011) | 1 | 1 | 0 | 0 | 0 | 0 | 0 | 1 | 1 | 2 | 2 | 2 | 0 | 0 | 2 | 2 | 10 | 12 |
| Sukhodolsky et al. (2013) | 1 | 2 | 1 | 0 | 0 | 1 | 0 | 2 | 1 | 2 | 2 | 0 | 2 | 0 | 1 | 5 | 10 | 15 |
| Wahlund et al. (2020) | 1 | 2 | 0 | 0 | 0 | 0 | 2 | 0 | 0 | 2 | 1 | 0 | 0 | 0 | 1 | 5 | 4 | 9 |
| Waldron et al. (2018) | 0 | 0 | 0 | 0 | 0 | 0 | 0 | 2 | 1 | 2 | 2 | 2 | 0 | 0 | 1 | 0 | 10 | 10 |

Appendix S2. Characteristics of RCTs named in the NICE guidelines for adolescent anxiety disorders

| Author, Year | Mean age | Age range | Target anxiety disorder | Was a previous SCED cited? |
| --- | --- | --- | --- | --- |
| Ahrens and Rexford (2002) | 16.4 | 15-18 | PTSD | No |
| Auslander et al. (2017) | 14.7 | 12-18 | PTSD | No |
| Barrett et al. (2004) | 13.56 | 7-17 | OCD | No |
| Barron et al. (2013) | 11.09 | 11-14 | PTSD | No |
| Barron et al. (2016) | 13.57 | 11-15 | PTSD | No |
| Beidel et al. (2007) | 11.18 | 7-17 | Social anxiety | No |
| Berger and Gelkopf (2009) | - | 9 to 14 | PTSD | No |
| Berger et al. (2012) | 12.8 | 11-13 | PTSD | No |
| Capaldi et al. (2016) | 15.3 | 13-18 | PTSD | No |
| Catani et al. (2009) | 12.3 | ‘8-14 | PTSD | No |
| Chen et al. (2014) | 14.5 | Adolescence | PTSD | No |
| Cohen et al. (2005) | 11.4 | 8-15 | PTSD | No |
| Crombach and Elbert (2015) | 17 | 11-23 | PTSD | No |
| Danielson et al. (2012) | 14.8 | 13-17 | PTSD | No |
| de Roos et al. (2017) | 13.4 | 8-18 | PTSD | No |
| Diehle et al. (2015) | 12.9 | 8-18 | PTSD | No |
| Ertl et al. (2011) | 11.65 | 12-25 | PTSD | No |
| Foa et al. (2013) | 15.3 | 13-18 | PTSD | No |
| Ford et al. (2012) | 14.7 | 13-17 | PTSD | No |
| Gilboa-Schechtman et al. (2010) | 14.05 | 12-18 | PTSD | No |
| Goldbeck et al. (2016) | 13.03 | ‘7-17 | PTSD | No |
| Herbert et al. (2009) | 14.3 | 12-17 | Social anxiety | No |
| Jaycox et al. (2009) | 11.5 | 11-13 | PTSD | No |
| Jensen et al. (2014) | 15.1 | 10-18 | PTSD | No |
| Jordans et al. (2010) | 12.7 | 11-14 | PTSD | No |
| Kaczkurkin et al. (2016) | 15.3 | 13-18 | PTSD | No |
| Kangaslampi et al. (2016) | 11.29 | 10-13 | PTSD | No |
| Kassam-Adams (2011) | 11.9 | 8-17 | PTSD | No |
| Kataoka et al. (2011) | 11 | 11-12 | PTSD | No |
| King et al. (2000) | 11.5 | 5-17 | PTSD | No |
| Layne et al. (2008) | 16 | 13-19 | PTSD | No |
| Masia Warner et al. (2007) | 15 | 14-16 | Social anxiety | No |
| McLean et al. (2015) | 15.3 | 13-18 | PTSD | No |
| McMullen et al. (2013) | 15.9 | 13-17 | PTSD | No |
| Meiser‐Stedman et al. (2017) | 13.3 | 8-17 | PTSD | No |
| O’Callaghan et al. (2013) | 15.8 | 12-17 | PTSD | No |
| O’Callaghan et al. (2014) | 13.16 | 7-18 | PTSD | No |
| Olivares-Olivares et al. (2008) | 15.3 | 14-18 | Social anxiety | No |
| Ooi et al. (2016) | 13.13 | 10-17 | PTSD | No |
| Pityaratstian et al. (2015) | 12.25 | 10-15 | PTSD | No |
| Punamäki et al. (2014) | 11.29 | 10-13 | PTSD | No |
| Qouta et al. (2012) | 11.29 | 10-13 | PTSD | No |
| Ruf et al. (2010) | 11.5 | 7-16 | PTSD | No |
| Shein‐Szydlo et al. (2016) | 15.05 | 12-18 | PTSD | No |
| Smith (2012) | 13.69 | 8-18 | PTSD | No |
| Spence et al. (2011) | 13.98 | 12-18 | Childhood anxiety | No |
| Stein et al. (2003) | 11 | 11-12 | PTSD | No |
| Swenson et al. (2010) | 13.88 | 10-17 | PTSD | No |
| Tillfors et al. (2011) | 16.5 | 15-21 | Social anxiety | No |
| Tol et al. (2014) | 12.29 | 8-17 | PTSD | No |
| Zandberg et al. (2016) | 15.3 | 13-18 | PTSD | No |
| Zehnder et al. (2010) | 11.8 | 7-16 | PTSD | No |

Note. Chen (2014) did not report an age range but instead said participants were “adolescence” with a mean age of 14.

**References**

Bernstein, E. R. (2010). Transportability of evidence-based anxiety interventions to a school setting: Evaluation of a modularized approach to intervention.

Chorpita, B. F., Yim, L., Moffitt, C., Umemoto, L. A., & Francis, S. E. (2000). Assessment of symptoms of DSM-IV anxiety and depression in children: A revised child anxiety and depression scale. *Behaviour Research and Therapy, 38*(8), 835-855.

Cowles, M., & Davis, J. (2017). Identifying, assessing and treating complicated post-traumatic stress disorder in adolescence: a single-case quasi-experimental design with clinical case report. *The Cognitive Behaviour Therapist, 10*.

Farrell, L. J., Oar, E. L., Waters, A. M., McConnell, H., Tiralongo, E., Garbharran, V., & Ollendick, T. (2016). Brief intensive CBT for pediatric OCD with E-therapy maintenance. *Journal of Anxiety Disorders, 42*, 85-94.

Farrell, L. J., Waters, A. M., Tiralongo, E., Mathieu, S., McKenzie, M., Garbharran, V., Ware, R. S., Zimmer‐Gembeck, M. J., McConnell, H., & Lavell, C. (2022). Efficacy of D‐cycloserine augmented brief intensive cognitive‐behavioural therapy for paediatric obsessive‐compulsive disorder: A randomised clinical trial. *Depression and Anxiety*.

Feather, J. S., & Ronan, K. R. (2009). Trauma‐focused CBT with maltreated children: A clinic‐based evaluation of a new treatment manual. *Australian Psychologist, 44*(3), 174-194.

Foa, E. B., Johnson, K. M., Feeny, N. C., & Treadwell, K. R. H. (2001). The Child PTSD Symptom Scale: A preliminary examination of its psychometric properties. *Journal of Clinical Child Psychology, 30*(3), 376-384.

Freeman, J., Flessner, C. A., & Garcia, A. (2011). The Children’s Yale-Brown Obsessive Compulsive Scale: reliability and validity for use among 5 to 8 year olds with obsessive-compulsive disorder. *Journal of Abnormal Child Psychology, 39*(6), 877-883.

Grefe, C. N. (2011). *Treating adolescent victims of physical and sexual abuse: A multiple baseline design*.

Hains, A. A., Davies, W. H., Behrens, D., & Biller, J. A. (1997). Cognitive behavioral interventions for adolescents with cystic fibrosis. *Journal of Pediatric Psychology, 22*(5), 669-687.

Heard, P. M., Dadds, M. R., & Conrad, P. (1992). Assessment and treatment of simple phobias in children: Effects on family and marital relationships. *Behaviour Change, 9*(2), 73-82.

Houghton, S., Alsalmi, N., Tan, C., Taylor, M., & Durkin, K. (2017). Treating comorbid anxiety in adolescents with ADHD using a cognitive behavior therapy program approach. *Journal of attention disorders, 21*(13), 1094-1104.

Kamphaus, R. W. (2014). Behavior Assessment System for Children, (BASC‐2). *The encyclopedia of clinical psychology*, 1-6.

Kane, M. T., & Kendall, P. C. (1989). Anxiety disorders in children: A multiple-baseline evaluation of a cognitive-behavioral treatment. *Behavior Therapy, 20*(4), 499-508.

Kendall, P. C. (1994). Treating anxiety disorders in children: results of a randomized clinical trial. *Journal of Consulting and Clinical Psychology, 62*(1), 100.

Kendall, P. C., Flannery-Schroeder, E., Panichelli-Mindel, S. M., Southam-Gerow, M., Henin, A., & Warman, M. (1997). Therapy for youths with anxiety disorders: A second randomized clincal trial. *Journal of Consulting and Clinical Psychology, 65*(3), 366.

Knight, R., Davies, R., Salkovskis, P. M., & Gregory, J. D. (2019). CBT with an adolescent with hoarding disorder—a single-case experimental design. *International Journal of Cognitive Therapy, 12*(2), 146-156.

March, J. S., Amaya-Jackson, L., Murray, M. C., & Schulte, A. (1998). Cognitive‐behavioral psychotherapy for children and adolescents with posttraumatic stress disorder after a single‐incident stressor. *Journal of the American Academy of Child and Adolescent Psychiatry, 37*(6), 585-593.

Morris, Z. S., Wooding, S., & Grant, J. (2011). The answer is 17 years, what is the question: understanding time lags in translational research. *Journal of the Royal Society of Medicine, 104*(12), 510-520.

Neil, N., Vause, T., Jaksic, H., & Feldman, M. (2017). Effects of group functional behavior-based cognitive-behavioral therapy for obsessive-compulsive behavior in a youth with autism spectrum disorder. *Child & Family Behavior Therapy, 39*(3), 179-190.

Ollendick, T. H. (1995). Cognitive behavioral treatment of panic disorder with agoraphobia in adolescents: A multiple baseline design analysis. *Behavior Therapy, 26*(3), 517-531.

Petoskey, A. V. C. (2015). A Single-case Neuropsychological Approach to Anxiety Reduction in an Adolescent with Autism Spectrum Disorder.

Rathus, J. H., Wagner, D., & Miller, A. L. (2015). Psychometric evaluation of the life problems inventory, a measure of borderline personality features in adolescents. *Journal of Psychology & Psychotherapy, 5*(4), 1-9.

Roberts‐Collins, C. (2016). A Case Study of an Adolescent With Health Anxiety and OCD, Treated Using CBT: Single‐Case Experimental Design. *Journal of Child and Adolescent Psychiatric Nursing, 29*(2), 95-104.

Sieberg, C. B., Flannery-Schroeder, E., & Plante, W. (2011). Children with co-morbid recurrent abdominal pain and anxiety disorders: results from a multiple-baseline intervention study. *Journal of Child Health Care, 15*(2), 126-139.

Silverman, W. K., & Nelles, W. B. (1988). The anxiety disorders interview schedule for children. *Journal of the American Academy of Child and Adolescent Psychiatry, 27*(6), 772-778.

Spielberger, C. D., Edwards, C. D., Montouri, J., & Lushene, R. (1973). State-trait anxiety inventory for children.

Sukhodolsky, D. G., Gorman, B. S., Scahill, L., Findley, D., & McGuire, J. (2013). Exposure and response prevention with or without parent management training for children with obsessive-compulsive disorder complicated by disruptive behavior: A multiple-baseline across-responses design study. *Journal of Anxiety Disorders, 27*(3), 298-305.

Suliman, S., Kaminer, D., Seedat, S., & Stein, D. J. (2005). Assessing post-traumatic stress disorder in South African adolescents: using the child and adolescent trauma survey (CATS) as a screening tool. *Annals of general psychiatry, 4*(1), 1-10.

Topper, M., Emmelkamp, P. M. G., Watkins, E., & Ehring, T. (2014). Development and assessment of brief versions of the Penn State Worry Questionnaire and the Ruminative Response Scale. *British Journal of Clinical Psychology, 53*(4), 402-421.

Vause, T., Neil, N., Jaksic, H., Jackiewicz, G., & Feldman, M. (2017). Preliminary randomized trial of function-based cognitive-behavioral therapy to treat obsessive compulsive behavior in children with autism spectrum disorder. *Focus on Autism and Other Developmental Disabilities, 32*(3), 218-228.

Wahlund, T., Jolstedt, M., Andersson, E., Vigerland, S., Perrin, S., Öst, L.-G., Högström, J., & Serlachius, E. (2020). Online cognitive behavior therapy for adolescents with excessive worry: a multiple baseline design feasibility study. *Mhealth, 6*.

Waldron, S. M., Maddern, L., & Wynn, A. (2018). Cognitive‐behavioural outreach for an adolescent experiencing social anxiety, panic and agoraphobia: A single‐case experimental design. *Journal of Child and Adolescent Psychiatric Nursing, 31*(4), 120-126.

Wei, C., Hoff, A., Villabø, M. A., Peterman, J., Kendall, P. C., Piacentini, J., McCracken, J., Walkup, J. T., Albano, A. M., & Rynn, M. (2014). Assessing anxiety in youth with the multidimensional anxiety scale for children. *Journal of Clinical Child and Adolescent Psychology, 43*(4), 566-578.
